# Association of Pregnancy With Coronavirus Cytokine Storm: Systematic Review and Meta-analysis

**DOI:** 10.2196/31579

**Published:** 2022-10-04

**Authors:** John Muthuka, Michael Kiptoo, Kelly Oluoch, Japheth Mativo Nzioki, Everlyn Musangi Nyamai

**Affiliations:** 1 Head Quaters Kenya Medical Training College Nairobi Kenya; 2 Department of Health Sciences South Eastern University of Kenya Kitui Kenya; 3 College of Health Sciences Jumeira University Dubai United Arab Emirates; 4 Department of Nursing Faculty of Clinical Sciences Kenya Medical Training College Nairobi Kenya

**Keywords:** COVID-19, pandemic, pregnancy, maternal health, cytokine, cytokine storm, immune response, infectious disease, coronavirus, respiratory, virus, pregnant

## Abstract

**Background:**

COVID-19 was first identified in Wuhan, China, in December 2019, spreading to the rest of the globe, becoming a pandemic. Some studies have shown an association between pregnancy status and severe COVID-19 with a cytokine storm, whereas others have shown contrasting results.

**Objective:**

The aim of this study was to examine the relationship between pregnancy status and the clinical COVID-19 severity characterized by the cytokine storm through a systematic review and meta-analysis.

**Methods:**

We searched the Google Scholar, PubMed, Scopus, Web of Science, and Embase databases to identify clinical studies suitable for inclusion in this meta-analysis. Studies reporting pregnancy status and comparing the COVID-19 severity cytokine storm outcome were included. COVID-19 severity characterized by a cytokine storm was described using parameters such as intensive care unit admission, invasive mechanical ventilation, mechanical ventilation, hospital admission, pro- and anti-inflammatory cytokine levels, consolidation on chest computed tomography scan, pulmonary infiltration, extreme fevers as characteristic of a cytokine storm, syndromic severity, higher neutrophil count indicative of a cytokine storm, and severe COVID-19 presentation.

**Results:**

A total of 17 articles including data for 840,332 women with COVID-19 were included. This meta-analysis revealed a correlation between positive pregnancy status and severe COVID-19 with a cytokine storm (random-effects model odds ratio [OR] 2.47, 95% CI 1.63-3.73; *P<.*001), with a cumulative incidence of 6432 (14.1%) and 24,352 (3.1%) among pregnant and nonpregnant women with COVID-19, respectively. The fixed-effects model also showed a correlation between pregnancy status and severe COVID-19 with a cytokine storm (OR 7.41, 95% CI 7.02-7.83; *P<.*001). Considerable heterogeneity was found among all pooled studies (*I²*=98%, *P<.*001). Furthermore, the updated analysis showed substantially low heterogeneity (*I²*=29 %, *P=*.19), and the funnel plot revealed no publication bias. The subanalysis between single-center and multicenter studies demonstrated similar heterogeneity (*I^2^*=72% and 98%, respectively). Sensitivity analysis on each subgroup revealed that pregnancy was significantly related to severe COVID-19 with a cytokine storm from single-center studies (fixed-effects model OR 3.97, 95% CI 2.26-6.95; *P*<.001) with very low heterogeneity (*I²*=2%, *P=*.42).

**Conclusions:**

Being pregnant is clearly associated with experiencing a severe course of COVID-19 characterized by a cytokine storm. The COVID-19 pandemic should serve as an impetus for further research on pregnant women diagnosed with COVID-19 to map out the salient risk factors associated with its severity.

**Trial Registration:**

PROSPERO CRD42021242011; https://www.crd.york.ac.uk/prospero/display_record.php?RecordID=242011.

## Introduction

Once considered to be an “immunosuppressed” state, pregnancy is associated with an immunological transformation, where the immune system is required to promote and support the pregnancy and growing fetus. When this protection is breached, as in a viral infection, this security is weakened and infection with microorganisms can then propagate and lead to negative outcomes such as preterm labor [1].

Pregnancy is considered a high-risk condition for COVID-19. Pregnant women are more likely to have an asymptomatic infection, accounting for 75% of SARS-CoV-2 infections during the pandemic. Even among those with symptoms, cough and fever are the main symptoms in 40% of cases, with breathing difficulty and myalgia being present in 21% and 19% of pregnant women, respectively. Severe COVID-19 usually occurs with infection in the second half of pregnancy, especially toward the end of the second trimester onward. Those at greatest risk of severe COVID-19 include women who have a higher-than-ideal BMI, those over the age of 35 years, and those who have chronic underlying conditions [2].

COVID-19 is an infectious disease caused by a newly discovered coronavirus (SAR2-CoV-2) that was first identified in Wuhan, China, in December 2019 [3]. COVID-19 subsequently rapidly spread across the world, causing a global pandemic. Between March 2020 and March 2021, this highly contagious disease infected over 25 million people worldwide and killed over 1 million patients, yielding a case fatality rate that varies between 0.7% and 12.7% (average 3.4%) [4].

Most people infected with the SARS-CoV-2 will experience mild to moderate respiratory illness and recover without requiring special treatment. Older people above the age of 58 years and those with underlying medical conditions such as cardiovascular disease, diabetes, chronic respiratory disease, and cancer are more likely to develop serious illnesses [5]. Further, infected patients experiencing cytokine storms present with fevers and shortness of breath, resulting in extreme difficulty breathing that ultimately requires ventilation assistance. Such severe presentations might also be related to pregnancy status [6].

Pregnant women who have COVID-19 appear more likely to develop respiratory complications requiring intensive care than women who are not pregnant [7]. Pregnant women are also more likely to be placed on a ventilator. Some research suggests that pregnant women with COVID-19 are also more likely to have a premature birth and cesarean delivery, and their babies are more likely to be admitted to a neonatal unit [8].

Pregnant women are a potentially highly vulnerable population due to anatomical, physiological, and immunological changes under the COVID-19 pandemic. Issues related to pregnancy with COVID-19 attracted widespread attention from researchers. A large number of articles were published aiming to elaborate on the clinical characteristics and outcomes of pregnant women infected with COVID-19 to provide evidence for management [9,10]. The existing data suggest that the overall prognosis of pregnancy with COVID-19 is promising when compared with that of other previous coronaviruses. However, there are still reports of notable maternal morbidity and mortality related to COVID-19 [9].

There are many unknowns for pregnant women during the COVID-19 pandemic. Clinical experience of pregnancies complicated with infection by other coronaviruses such as severe acute respiratory syndrome (SARS) and Middle Eastern respiratory syndrome (MERS) indicated that pregnant woman should be considered to be particularly vulnerable to severe SARS-CoV-2 infection. Physiological changes during pregnancy have a significant impact on the immune system, respiratory system, cardiovascular function, and coagulation [11].

Given divergent findings in the existing literature, we systematically reviewed English-language studies to investigate whether pregnancy was associated with a more severe clinical course of COVID-19. Specifically, the aim of this study was to establish if pregnancy status is associated with COVID-19 severity characterized by a cytokine storm.

## Methods

### Design

All guidelines listed in the PRISMA (Preferred Reporting Items for Systematic Reviews and Meta-Analyses) statement were followed in performing this meta-analysis [12]. For this systematic review and meta-analysis, data were pooled from observational studies, including cohort, case-control, cross-sectional, and similar viable case studies. The study is registered in PROSPERO (CRD42021242011).

### Search Strategy

We performed a simple search in the Google Scholar, PubMed, Scopus, Web of Science, and Embase databases to identify observational studies suitable for inclusion with the following search terms: “COVID-19” OR “SARS-COV-2” OR “novel coronavirus (CoV)” AND “pregnant” OR “gestation” AND “clinical features” OR “characteristic” AND “severity” OR “severe.” Studies were restricted to those published in English from March 2020 to March 2021.

### Inclusion and Exclusion Criteria

Inclusion criteria were as follows: (1) studies that examined women within reproductive age and diagnosed with COVID-19 according to World Health Organization (WHO) criteria; (2) observational, cross-sectional, prospective, or retrospective studies; (3) studies that compared pregnant women to nonpregnant women with severe COVID-19 characterized by a cytokine storm; (4) studies evaluating the clinical prognosis in pregnancy and the immunological profile at any gestation stage, examining the proinflammatory response in COVID-19 and a severe cytokine storm as the hallmark outcome.

Exclusion criteria were as follows: (1) unrelated, duplicated, and missing information answering our research question; (2) non-English-language studies; (3) case reports/series; (4) reviews; (5) editorials; (6) studies lacking a full text (unavailable or not yet published); (7) articles without a DOI; and (8) studies with small sample sizes (<50 patients) because of low statistical power.

Notably, we included preliminary findings published as preprints given that the phenomenon in question remains very grey in the public domain and thus we presumed inclusion of such reports would be of value in converging relevant data and information.

### Data Extraction

Both adjusted and nonadjusted data among pregnant versus nonpregnant cases were extracted to identify the most relevant confounding factors to be used in the analysis by subsequent pooling. One reviewer (JM) scanned study titles and abstracts obtained via an initial database search and included relevant articles in a secondary pool. Next, two independent reviewers (MK and KO) evaluated the full texts of these articles to determine whether they met the study inclusion criteria. Any disputes were resolved by discussion and negotiation with a fourth reviewer (EN). Only studies agreed upon by all reviewers were included in the final analysis.

The following data were obtained from all studies: title, first author, publication year, location, sample size, age (median), pregnancy status (pregnant or nonpregnant), and severe COVID-19 cytokine storm presentation. The analysis was then performed to determine whether the pregnant group was more likely to develop severe COVID-19 characterized by a cytokine storm.

### Risk of Bias (Quality) Assessment

The National Institutes of Health tool for observational and cross-sectional studies [13] was used for methodological quality assessment. Two to three reviewers independently assessed the quality of the studies, and the scores were added to the data extraction form before inclusion in the analysis to reduce the risk of bias. To evaluate the risk of bias, the reviewers rated each of the 14 items into qualitative variables: yes, no, or not applicable. An overall score was calculated by adding the scores of all items with yes=1 and no or not applicable=0. A score was given for every paper, resulting in a classification of poor (score 0-5), fair (score 6-9), or good (score 10-14). Data were checked by reviewers who did not perform the data extraction or each reviewer was assigned an article that they had not extracted data from in previous steps; however, in rare instances, some reviewers extracted data and performed the quality assessment for the same article.

### Statistical Analyses

Review Manager 5.4.1 was used to calculate odds ratios (ORs) with 95% CIs, which are depicted using forest plots. Quantitative variables are summarized in terms total numbers and percentages. The OR of a severe COVID-19 cytokine storm among pregnant and nonpregnant women was calculated. Heterogeneity was evaluated with the Cochran Q statistic and Higgins test. The Higgins test uses a fixed-effects model when the heterogeneity is <50% and a random-effects model when the heterogeneity is >50%. When heterogeneity was detected, a sensitivity adjustment was made to determine its source. This procedure was performed by leaving a study out of the analysis one at a time, with the fixed-effects model applied after excluding heterogeneity. Subgroup, cumulative analyses, and metaregression were used to test whether or not the results are consistent and to investigate the effect of confounders on the outcome (cytokine storm) and elucidate the best predictors in pregnancy status among women with COVID-19. Publication bias was evaluated using the Cochrane Risk of Bias tool.

## Results

### Included Articles and Quality Assessment

The initial search of international databases using the keywords described above yielded 221 articles. After excluding 70 duplicate articles, 151 articles remained. When article titles and abstracts were evaluated for appropriateness, 29 articles ultimately met the inclusion criteria. In addition, 12 articles not meeting the inclusion criteria were excluded after full-text review. A total of 17 articles met the inclusion criteria [7,14-29]. Multimedia Appendix 1 shows the PRISMA flow diagram of the study selection procedure.

### Features of the Included Studies

The 17 included studies provided data for 840,417 women with COVID-19 [7,14-29] (Table 1). According to the Centers for Disease Control and Prevention reporting guidelines for COVID-19 diagnosis [30], 85 patients whose specific parameters related to the severity of COVID-19 defined according to cytokine storm status were reported as “unknown” or not tabulated were excluded from the final analysis, yielding a final group of 840,332 patients with 45,571 (5.42%) pregnant women and 794,761 (94.58%) nonpregnant women. Among the pregnant women, 14.1% (6432/45,571) had cytokine storm events reported, compared to only 3.1% (24,352/794,761) of the nonpregnant women. The cumulative incidence of a cytokine storm from all studies ranged from 0.4% to 90.7% (average 36.26%).

**Table 1:** Features of the studies included in the meta-analysis.

Reference	Location of patients	Study design	Parameter of comparison on COVID-19 severity with cytokine storm	Events in pregnant women/ total in cohort	Events in nonpregnant women/total in cohort	Cumulative incidence of severe COVID-19 defined by cytokine storm, n (%)
Badr et al [14]	France and Belgium	CC^a^, MC^b^	ICU^c^ versus no ICU admission	58/83	17/107	75 (39%)
Westgren and Acharya [15]	New York	R^d^, O^e^, MC	ICU versus no ICU admission	8/82	50/332	58 (14%)
CDC^f^ [16]	United States	P^g^, C^h^, MC	ICU plus mechanical ventilation versus no ICU admission with mechanical ventilation	2583/8200	15,840/316,800	18,423 (5.7%)
Cheng et al [17]	Wuhan, China	R, SC^i^	Higher versus lower level of inflammation markers	0/31	1/80	1 (0.9%)
Collin et al [23]	Sweden	R, MC	Invasive mechanical ventilation versus no invasive mechanical ventilation	7/13	29/40	36 (68%)
Ellington et al [7]	United States	R, O MC	ICU with mechanical ventilation versus no ICU with mechanical ventilation	2587/8207	4840/83,205	7427 (8%)
Liu et al [24]	Wuhan, China	R, CC, SC	Consolidation on chest CT^j^ versus no consolidation on chest CT	20/21	16/19	36 (90%)
Martinez-Portilla et al [25]	Mexico	R, MC	ICU/death versus non-ICU/death	752/5183	446/5183	1198 (12%)
Yin et al [26]	China	R, C, SC	Severe or critical COVID-19 characterized by higher levels of inflammatory indices of cytokine storm versus moderate COVID-19	19/31	11/35	30 (46%)
Mohr-Sasson et al [27]	Fuyang, China	R, C, SC	High versus low fevers	3/11	15/25	18 (50%)
Molteni et al [28]	United Kingdom, Sweden, and United States	P, O, MC	Syndromic severity versus nonsyndromic severity	87/140	1508/2515	1595 (60%)
Oakes et al [18]	Wuhan, China	R, C, SC	Hospital admission versus nonadmission	7/22	17/240	24 (9%)
Qiancheng et al [19]	Wuhan, China	R, SC	Nonsevere versus severe	2/28	1/54	3 (9.8%)
Wang et al [20]	Wuhan, China	R, SC	COVID-19 manifestations on chest CT versus no manifestations	22/30	42/42	64 (89%)
Wei et al [21]	Wuhan, China	R, SC	Higher versus lower neutrophil count as indicative of cytokine storm	15/17	24/26	39 (91%)
Xu et al [22]	Wuhan, China	R, SC	Pulmonary infiltration versus no pulmonary infiltration	17/34	3/30	20 (31%)
Zambrano et al [29]	United States	R, MC	Severe COVID-19–associated illness versus mild to moderate illness	245/23,434	1492/386,028	1737 (0.4%)

^a^CC: case-control.

^b^MC: multicenter.

^c^ICU: intensive care unit.

^d^R: retrospective.

^e^O: observational.

^f^CDC: Centers for Disease Control and Prevention.

^g^P: prospective.

^h^C: cross-sectional.

^i^SC: single-center.

^j^CT: computed tomography.

The main outcome of this meta-analysis was the possible association of pregnancy with severe COVID-19 characterized by a cytokine storm, which was indicated by a specific prognosis and event. The parameters used for assessment of COVID-19 severity were intensive care unit (ICU) admission in three studies; ICU plus mechanical ventilation in two studies; higher levels of inflammatory response markers in three studies; severe COVID-19 presentation in two studies; and consolidation on chest computed tomography scan, pulmonary infiltration, extreme fever as a characteristic of a cytokine storm, syndromic severity, hospital admission, invasive mechanical ventilation, and higher neutrophil count indicative of a cytokine storm in one study each. The study designs included retrospective (n=15, 6 multicenter and 9 single-center studies) and prospective (n=2, both multicenter). A summary of the studies included in the meta-analysis is provided in Table 1.

We assessed the quality of the included observational studies based on a modified version of the Newcastle-Ottawa Scale (NOS), which consists of 8 items with 3 subscales, and the total maximum score of these 3 subsets is 9. We considered a study that scored ≥7 to be a high-quality study since a standard criterion for what constitutes a high-quality study has not yet been universally established. The 17 studies assessed generated a mean value of 6.47, indicating that the overall quality was moderate (NOS score range 5-8), as detailed in Table 2.

**Table 2:** Newcastle-Ottawa scale for quality assessment and risk of bias.

Study	Year	Case selection (maximum 4)	Comparability (maximum 2)	Exposure/outcome (maximum 3)	Total score
Badr et al [14]	2020	3	2	2	7
Westgren and Acharya [15]	2020	3	2	1	6
CDC^a^ [16]	2020	4	2	2	8
Cheng et al [17]	2020	3	1	2	6
Collin et al [23]	2020	4	1	2	7
Ellington et al [7]	2020	3	2	3	7
Liu et al [24]	2020	3	1	2	6
Martinez-Portilla et al [25]	2020	3	1	2	6
Yin et al [26]	2020	3	2	2	7
Mohr-Sasson et al [27]	2020	3	1	1	5
Molteni et al [28]	2020	3	1	2	6
Oakes et al [18]	2020	3	2	2	7
Qiancheng et al [19]	2020	3	1	2	6
Wang et al [20]	2020	2	2	2	6
Wei et al [21]	2020	3	1	3	7
Xu et al [22]	2020	3	2	2	6
Zambrano et al [29]	2020	3	1	3	7

^a^CDC: Centers for Disease Control and Prevention.

### Pregnancy Status and COVID-19 Severity Characterized by a Cytokine Storm

**Figure 1:**
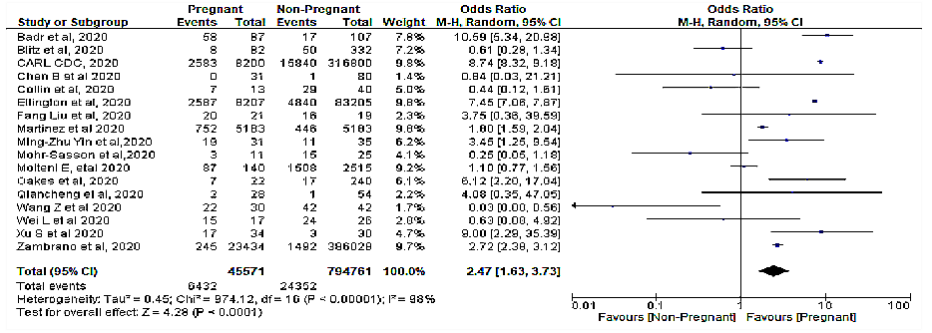
A forest plot of meta-analysis between pregnancy status and severe COVID-19 with cytokine storm.

**Figure 2:**
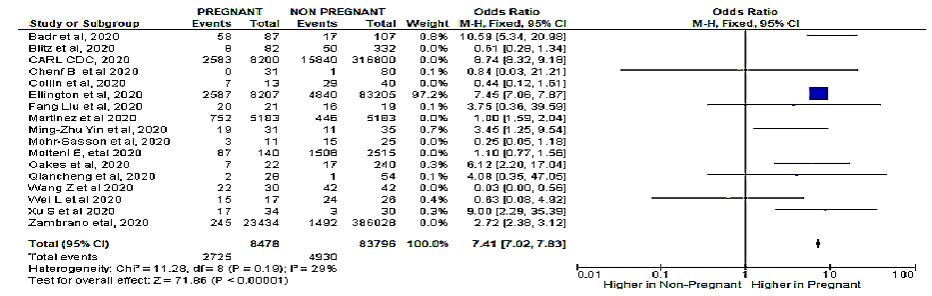
Forest plot of the association of pregnancy with severe COVID-19 characterized by a cytokine storm with the fixed-effects model.

**Figure 3:**
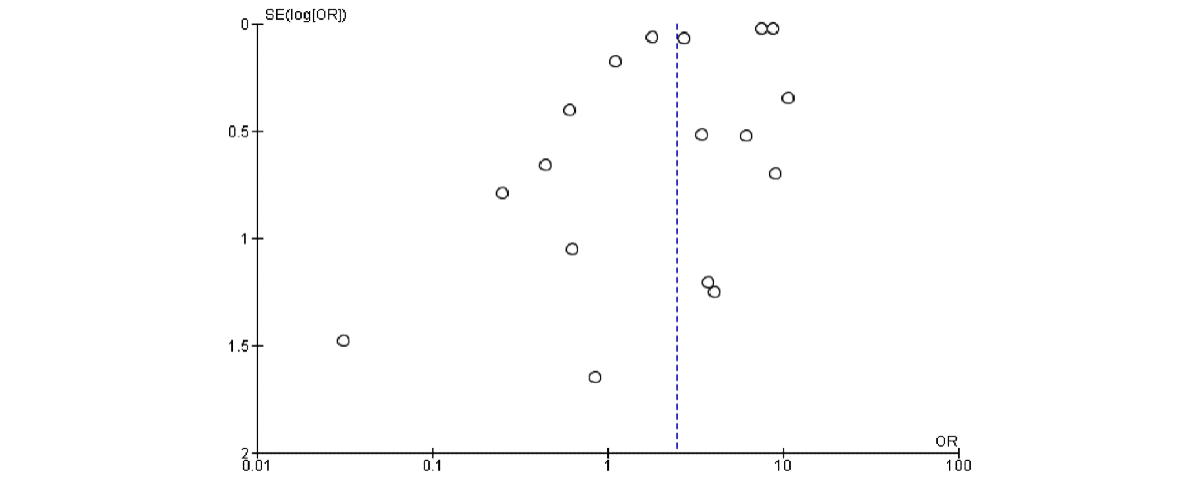
Funnel plot evaluating publication bias. OR: odds ratio.

**Figure 4:**
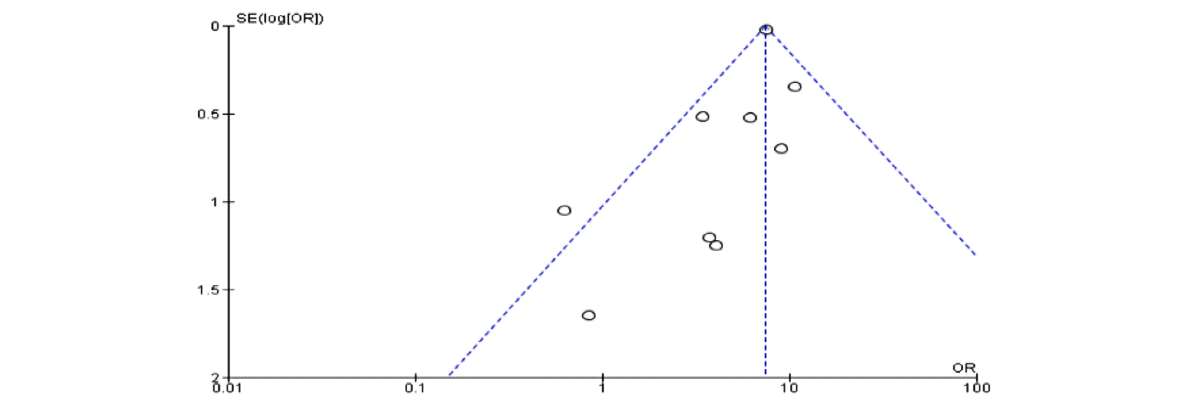
Funnel plot revealing no publication bias in the updated analysis. OR: odds ratio.

The meta-analysis revealed a significant association between pregnancy status and severe COVID-19 characterized by a cytokine storm (Table 3). A sensitivity analysis was performed to explore the impact of excluding or including studies in the meta-analysis based on sample size, methodological quality, and variance. After removing eight studies (n=748,058 patients) [7,15,16,23,25,27,28,31] accounting for major causes of heterogeneity, a total of 92,274 patients were left for analysis in the remaining studies. Figure 1 and Figure 2 respectively show a shift from the random-effects model (OR 2.47, 95% CI 1.63-3.73; *P<.*001) to the fixed-effects model (OR 7.41, 95% CI 7.02-7.83; *P<.*001), revealing that pregnancy was significantly associated with severe COVID-19 characterized by a cytokine storm. Furthermore, this updated analysis showed substantially low heterogeneity (*I²*=29%, *P=*.19). Figure 3 shows a funnel plot evaluating publication bias, which revealed considerable heterogeneity between all pooled studies (*I²*=98%, *P<.*001). Figure 4 shows a funnel plot revealing no publication bias for the updated analysis.

**Table 3:** Events (cytokine storm) in pregnant and nonpregnant women.

Studies	Pregnant with COVID-19			Nonpregnant with COVID-19	
	Patients, N	Events, n (%)	Patients, N		Events, n (%)
Badr et al [14]	87	58 (66.7)	107		17 (15.9)
Westgren and Acharya [15]	82	8 (9.8)	332		50 (15.1)
CDC [16]	8200	2583 (31.5)	316,800		15,840 (5.0)
Cheng et al [17]	31	0 (0)	80		1 (1.3)
Collin et al [23]	13	7 (53.8)	40		29 (72.5)
Ellington et al [7]	8207	2587 (31.5)	83205		4840 (5.9)
Liu et al [24]	21	20 (95.3)	19		16 (84.2)
Martinez-Portilla et al [25]	5183	752 (14.5)	5183		446 (8.6)
Yin et al [26]	31	19 (61.3)	35		11 (31.4)
Mohr-Sasson et al [27]	11	3 (27.2)	25		15 (60.0)
Molteni et al [28]	140	87 (62.1)	2515		1508 (59.9)
Oakes et al [18]	22	7 (31.8)	240		17 (7.1)
Qiancheng et al [19]	28	2 (7.14)	54		1 (1.9)
Wang et al [20]	30	22 (73.3)	42		42 (100.0)
Wei et al [21]	17	15 (88.2)	26		24 (92.3)
Xu et al [22]	34	17 (50.0)	30		3 (10.0)
Zambrano et al [29]	23,434	245 (1.1)	386,028		1492 (0.4)

### Subgroup Analysis and Investigation of Heterogeneity

**Figure 5:**
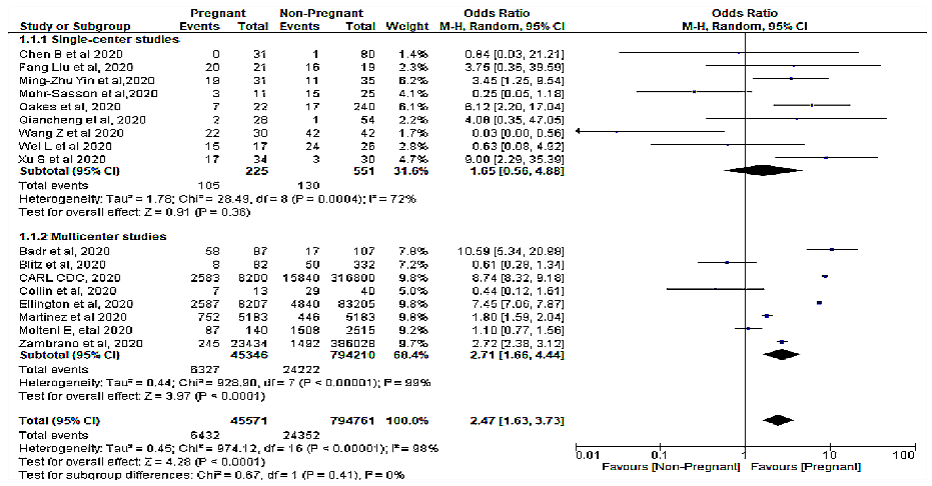
Subgroup analysis according to single-center or multicenter study designs showing similarly high heterogeneity as the full meta-analysis.

**Figure 6:**
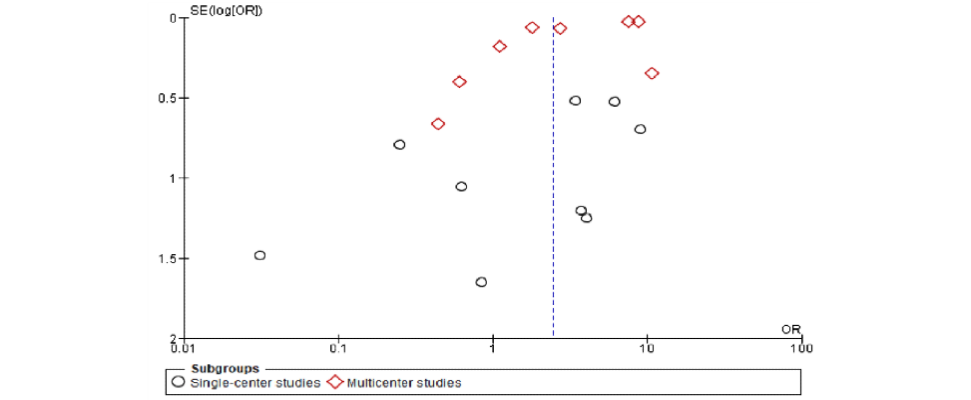
Funnel plot of the subgroup analysis-single-center and multicenter studies.

Heterogeneity in the pooled effect estimates was considerably high for all 17 studies, contributed by 748,058 out of 840,332 (89.02%) evaluated subjects, and thus it was necessary to perform subgroup analyses to identify possible variables or characteristics moderating the results obtained. Subgroup analysis was performed according to whether it was a multicenter study, including 879,556 patients, or a single-center study with 776 patients. Figures 5 and 6 show that subgroup analysis still showed high heterogeneity (*I^2^*=72%). The test for the overall effect for single-center studies (*Z*=0.91, *P*=.36; *I^2^*=98) and multicenter studies (*Z*=3.97, *P*<.001) showed no significance difference (χ_1_^2^=0.67, *P*=.41; *I²*=0%). This prompted further sensitivity analysis on each subgroup to ascertain the group that was most strongly associated with heterogeneity.

**Figure 7:**
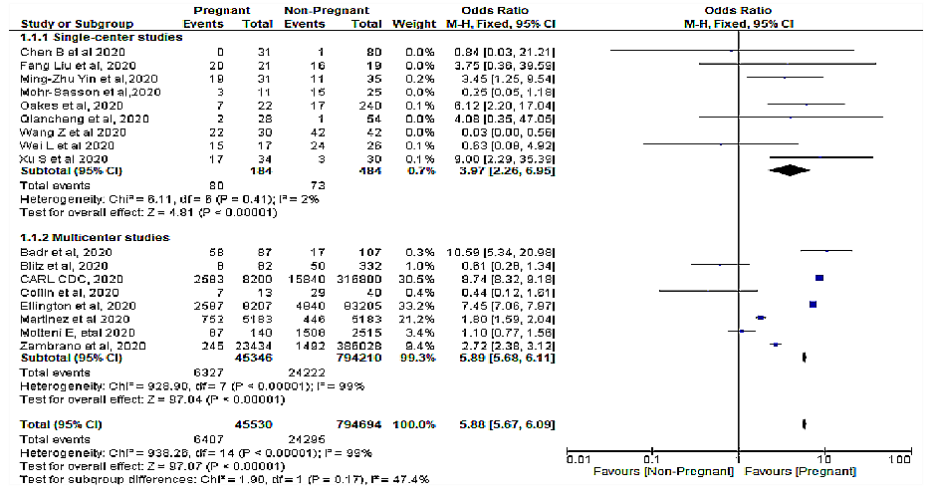
Sensitivity analysis on independent subgroups.

**Figure 8:**
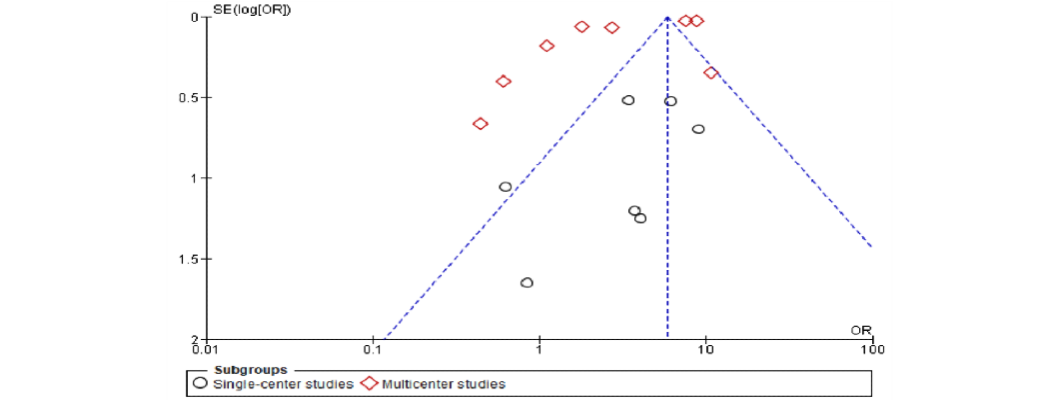
Funnel plot of sensitivity analysis on independent subgroups (single-center and multicenter) to evaluate publication bias.

Figure 7 shows the sensitivity analysis on independent subgroups. In single-center studies, elimination of studies that caused the major heterogeneity ([27] and [31]; n=108) revealed that pregnancy was significantly related to severe COVID-19 with a cytokine storm represented by 668 patients (fixed-effects model OR 3.97, 95% CI 2.26-6.95; *P*<.001), with this updated analysis showing substantially low heterogeneity (*I²*=2%, *P=*.42). In multicenter studies, subsequent removal of any one study did not change the heterogeneity from its original value (*χ^2^*_7_=928.90, *P*<.001; *I²*=99%), demonstrating that multicenter studies were the main cause of heterogeneity and this was similar to the overall heterogeneity of the combined groups (fixed-effects model heterogeneity *χ^2^*_14_=938.26, *P*<.001; *I²*=99%), with the test for subgroup differences being insignificant (*χ*^2^_1_=1.9, *P*=.17; *I²*=47.4%). Figure 8 shows a funnel plot similarly demonstrating that multicenter studies were associated with heterogeneity with only one study demonstrating homogeneity.

## Discussion

This review established that pregnancy is associated with an experience of severe COVID-19 characterized by a cytokine storm. Heterogeneity analysis revealed that the pooled effect estimate was considerably high considering all 17 included studies, contributed by 89% of the total patients evaluated. Further, sensitivity analysis on each subgroup indicated that single-center studies were more homogeneous in comparison to multicenter studies.

This meta-analysis included 17 studies and revealed that pregnant women had a significantly increased risk for severe COVID-19 characterized by a cytokine storm. Previous research has indicated a similar association [32,33]. Additionally, another meta-analysis reported the outcome of coronavirus spectrum infections (SARS, MERS, and COVID-19) during pregnancy, showing that COVID-19 disease severity increased during gestation [34]. This analysis adds to the extensive consensus in the literature, which should motivate more studies examining pregnancy status as a possible predictor of severe COVID-19 characterized by a cytokine storm.

Prior studies have reported results that contrast with those presented here, namely a lack of significant difference between pregnant and nonpregnant women diagnosed with COVID-19 in terms of disease severity [35,36]. In addition, a previous meta-analysis [37] failed to find a relationship between being pregnant and severe COVID-19 in 24 studies including pregnant women, and another meta-analysis indicated that COVID-19 infection during pregnancy most likely had a clinical presentation and severity resembling those in nonpregnant adults [38]. Moreover, a meta-analysis demonstrated similar trends in disease severity between pregnant people and the general population [39]. Further, two more studies showed no feasible differences in the clinical presentation of COVID-19 between pregnant and nonpregnant women [40,41]. Of concern, neither of the meta-analyses mentioned above [37,38] included an assessment of publication bias or study quality. As such, these studies should be considered as only a preliminary quest. Hence, the present systematic meta-analysis offers a more detailed view as it covers 17 studies from diverse regions capturing both single and multiple centers. The heterogeneity was high, and after sensitivity adjustments to eliminate studies largely responsible for the heterogeneity, the association of COVID-19 severity with pregnancy was revealed with substantially low heterogeneity. Furthermore, the subgroup analysis after performing the sensitivity test in each specified subgroup (multicenter or single-center studies) showed a clear significant association between being pregnant and developing severe COVID-19 characterized by any specific parameter of a cytokine storm in single-center studies. Therefore, severe COVID-19 was observed to be almost 4 times (OR 3.97, 95% CI 2.26-6.95; *P*<.001) more frequent in pregnant women. Some previous studies, including some meta-analyses [39,42-46], support the current findings.

A recent meta-analysis revealed that SARS-CoV-2 infection may not manifest as mild symptoms during pregnancy [47]. Interestingly, this meta-analysis showed that 40 patients developed pneumonia, bilateral in most cases, with a 46.2% rate of hospitalization and 4 patients required ICU admission. The same study found a higher rate of severe forms of COVID-19, even when compared to nonpregnant women with the same baseline characteristics [47]. This appears to be because, during the gestation period, pregnant women face proinflammatory episodes that mimic the trends of a cytokine storm in the case of severe COVID-19. This has been demonstrated in recent studies where specific immune cells, especially neutrophils, and other biomarkers have been highlighted as essential effector cells in the development of COVID-19 [48-51]. In addition, pregnancy has been reported to increase the progression of COVID-19 [52]. There is growing evidence to support the WHO’s statements that pregnant women are at a higher risk of developing severe COVID-19–related symptoms and possible mortality [53-56]. Indeed, pregnancy has been found to worsen the morbidity of COVID-19, and this effect becomes more prominent as pregnancy advances [57].

The association between pregnancy and illness severity due to other respiratory viruses such as MERS has been investigated previously. In one study, the case fatality (25%), ICU admission (50%), and mechanical ventilation (33%) rates were increased in the pregnant population compared with those of the nonpregnant population (20%) [58], which may be related to abnormal immune responses in pregnancy. Additionally, pregnancy may propagate respiratory infections and increase the risk of hospitalization [59]. Another study demonstrated that complications of severity with other acute respiratory distress syndromes are enhanced in pregnancy [11]. As a result, adverse effects on the pregnant woman’s lungs may aggravate the symptom severity of viral infections.

The novel SARS-CoV-2 virus uses angiotensin-converting enzyme 2 (ACE2) receptor in the lungs to enter cells and cause infection. ACE2 expression and activity are enhanced during pregnancy, and transient ACE2 overexpression and its increased activity during pregnancy may be important in modulating systemic as well as local hemodynamics in the uteroplacental unit [60,61]. ACE2 upregulation may increase infectiousness and therefore infection severity risk, as the SARS-CoV-2 virus uses this receptor for host entry. Paradoxically, ACE2 upregulation has also been reported to be a protective factor against acute lung injury [62].

In one recent study, *ACE2* gene expression was found to be upregulated in cells specific to the maternal-fetal interface [63], thereby suggesting a mechanism by which the risk for severe COVID-19 increases in pregnancy. A role of ACE2 in COVID-19 pathophysiology has also been demonstrated, including factors influencing ACE2 expression and activity in relation to COVID-19 severity [64]. Thus, the potential impact of ACE2 expression and thus SARS-CoV-2 entry into the host in pregnancy should be further investigated [65].

The cytokine storm phenomenon has received substantial research attention recently because of the COVID-19 pandemic. Although more and more information is accumulating daily, the cytokine storm seems to be at least part of the reason that some people develop life-threatening symptoms from COVID-19. Hyperinflammatory cytokine storms in many patients with severe symptomatic cases of COVID-19 may be rooted in an atypical response to SARS-CoV-2 by dysfunctional mast cells, in a condition known as mast cell activation syndrome, rather than the typical response by normal mast cells [66]. This may be explained by systemic and chronic inflammation, diminished respiratory function and capacity, and chronic obstructive pulmonary disease–related respiratory failure in some patients. Some findings indicated an association of pro- and anti-inflammatory cytokines that play crucial roles in the development and function of preeclampsia [67]. Given this, pregnancy itself and pro- and anti-inflammatory cytokines should be considered together as a single risk factor for severe COVID-19 among pregnant women diagnosed with the novel coronavirus.

Another critical area of concern is that the cytokine storm is a critical contributor to mortality in some patients with severe COVID-19. In these patients, the levels of proinflammatory cytokines such as interleukin (IL)-1, IL-2, IL-6, IL-8, IL-17, interferon (IFN)-γ, and tumor necrosis factor (TNF)-α are elevated, which affect the patient’s clinical symptoms and severity in the general population [68]. In pregnancy, IFNs and cytokines play important roles in the immune responses promoting healthy pregnancy as well as congenital disorders and complications [69], similar to those activated during a COVID-19 cytokine storm, including TNF-α [70]. Increased levels of INF-γ, luteinizing hormone, and prolactin have been identified as the underlying cause for recurrent pregnancy losses; thus, these factors not only amplify the severity of the cytokine storm in COVID-19 but also consequentially result in adverse pregnancy outcomes [70]. This potential interaction should be clarified with future clinical research.

Several factors limit the interpretation of the present study. First, the vast majority of studies included in the meta-analysis were retrospective epidemiological studies conducted in the United States and China, with limited studies from other regions. Second, some of the included studies did not distinguish the age range of the participants as well as the stage of the gestation period. Third, COVID-19 severity as assumed to be characterized by a cytokine storm relied on different parameters of clinical implications such as the levels of inflammatory cytokines, invasive mechanical ventilation, and ICU admission. Given these limitations, caution should be exercised when interpreting the current findings for more valid clinical practice. Future studies may respond to these issues by defining disease severity more clearly and by obtaining more detailed information on the associated inflammatory cytokines defining the COVID-19 cytokine storm.

Multiple factors are responsible for recurrent pregnancy loss, although an altered cytokine profile is known to be a major contributor, especially in the early stages of gestation. Similarly, exposure to high maternal proinflammatory cytokine concentrations in early pregnancy might play a role in several adverse effects for either the woman or infant. Thus, women expecting a pregnancy should be screened to assess the cytokine profile even prior to conception whenever possible to avoid pregnancy loss and to improve their health and social well-being, as abnormal cytokine levels could aggravate COVID-19 severity.

Finally, the interactions between the inherent inflammatory cytokines and cytokine storm due to COVID-19 should also be further examined and clarified. In addition, clinicians should pay more attention to the history of pregnancy-related altered immune responses of COVID-19 patients. Further research may aim to determine the mechanisms that drive or decrease this risk of severity by a within–pregnant population study approach.

This meta-analysis revealed that pregnancy is significantly associated with increased COVID-19 symptom severity defined by a cytokine storm. The SARS-CoV-2 epidemic should serve as an impetus for further research on pregnant women diagnosed with COVID-19, and to map out salient risk factors associated with its severity with an aim of maintaining a good pregnancy outcome and possibly evading an adverse COVID-19 clinical prognosis.

## Appendix

Multimedia Appendix 1 PRISMA (Preferred Items for Systematic Reviews and Meta-Analyses) flow diagram.

## Abbreviations

ACE2: angiotensin-converting enzyme 2
ICU: intensive care unit
IFN: interferon
IL: interleukin
MERS: Middle East respiratory syndrome
NOS: Newcastle-Ottawa Scale
OR: odds ratio
PRISMA: Preferred Reporting Items for Systematic Reviews and Meta-Analyses
SARS: severe acute respiratory syndrome
TNF: tumor necrosis factor
WHO: World Health Organization
