# Supplementary figures and images for: Association of Pregnancy With Coronavirus Cytokine Storm: Systematic Review and Meta-analysis
Source: JMIR Pediatr Parent. 2022 Oct 4;5(4):e31579. doi: 10.2196/31579 (PMC9534275; doi:10.2196/31579)

## PRISMA study flow diagram of study selection procedures

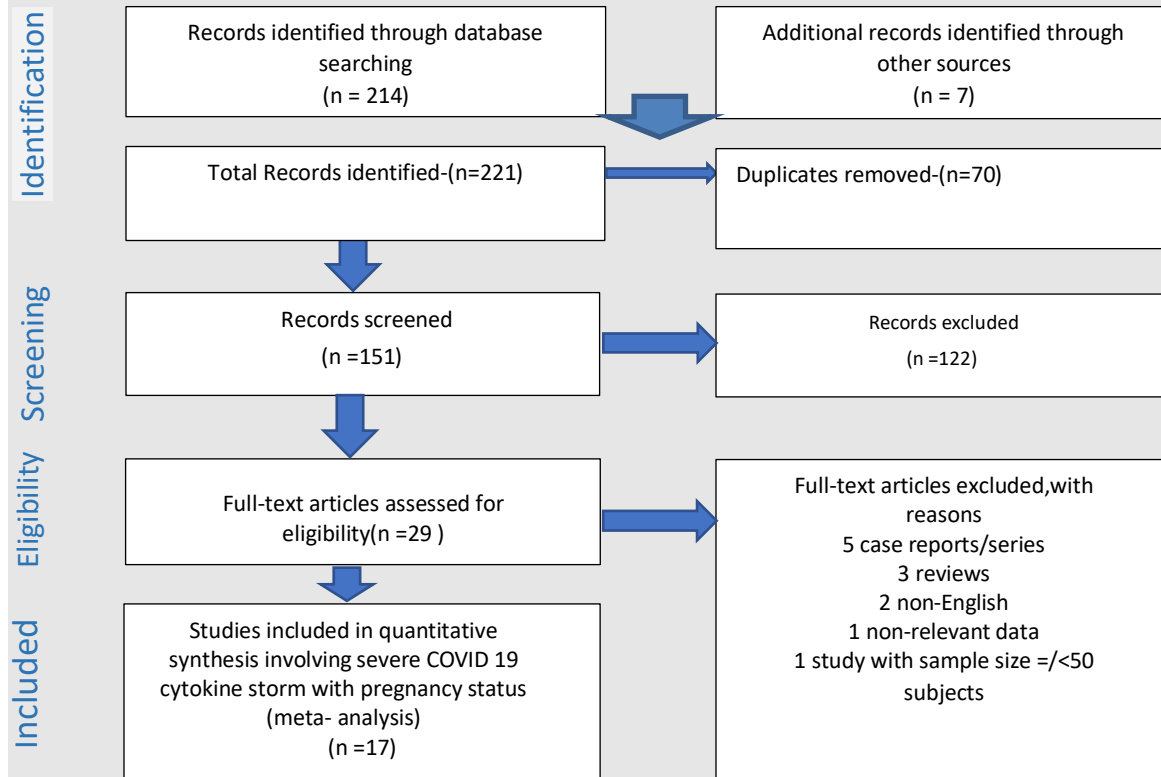

Supplement: Multimedia Appendix 1 [file pediatrics_v5i4e31579_app1.pdf]
